# Can we Replace Arterial Blood Gas Analysis by Pulse Oximetry in Neonates with Respiratory Distress Syndrome, who are Treated According to INSURE Protocol?

**Published:** 2015-05

**Authors:** Pedram Niknafs, Elahe Norouzi, Bahareh Bahman Bijari, Mohammad Reza Baneshi

**Affiliations:** 1 Division of Neonatology, Afzalipour Medical Center, Kerman University of Medical Sciences, Kerman, Iran;; 2 Research Center for Modeling in Health, Institute for Future Studies, Kerman University of Medical Sciences, Kerman, Iran

**Keywords:** Infant, Respiratory distress syndrome, Oximetry, Blood gaz analysis

## Abstract

Neonates with respiratory distress syndrome (RDS), who are treated according to INSURE protocol; require arterial blood gas (ABG) analysis to decide on appropriate management. We conducted this study to investigate the validity of pulse oximetry instead of frequent ABG analysis in the evaluation of these patients. From a total of 193 blood samples obtained from 30 neonates <1500 grams with RDS, 7.2% were found to have one or more of the followings: acidosis, hypercapnia, or hypoxemia. We found that pulse oximetry in the detection of hyperoxemia had a good validity to appropriately manage patients without blood gas analysis. However, the validity of pulse oximetry was not good enough to detect acidosis, hypercapnia, and hypoxemia.

## Introduction

In neonates with birth weight <1500 grams, when the clinical diagnosis of respiratory distress syndrome (RDS) is made based on the respiratory symptoms and confirmatory studies (e.g. blood gas analysis, chest x-ray, etc.), the appropriate management is to place the patient under nasal continuous positive airway pressure (NCPAP). If respiratory distress increases, surfactant is administered according to INSURE protocol^1^ (transient intubation, surfactant administration, rapid extubation to NCPAP^2^). These infants require an umbilical artery catheter to obtain arterial blood gas (ABG) every 30 minutes to 4 hours for accurate monitoring of gas exchange.^3^

Arterial blood gas analysis is the gold standard by which the adequacy of oxygenation and ventilation are assessed.^1^ Although umbilical catheterization is safe and well tolerated in most infants, it is associated with serious complications which could be life threatening.^3-5^

Pulse oximetry is a technique that indirectly determines oxygenation in a continuous noninvasive manner. The percentage of saturated hemoglobin is calculated from the difference between light frequencies of the pulsatile flow as it passes beneath the sensor at distal extremity of the infant.^3^^,^^4^

We did not find any study regarding the validity of pulse oximetry as a bedside monitoring in the assessment of neonates with birth weight lower than 1500 grams with RDS. Therefore, we conducted this study to investigate the diagnostic value of pulse oximetry in detecting acidosis, hypercapnia, hypoxemia, and hyperoxemia. 

## Patients and Methods

This was a prospective diagnostic test study (carried out at the Afzalipour Medical Center in Kerman-Iran, from October 2011 to March 2012) on 30 preterm infants with moderately severe RDS weighing less than 1500 grams at birth.^6^ Neonates in whom surfactant had been introduced as INSURE protocol met the criteria for the study. These infants were placed under bubble NCPAP with FiO_2_ of 40-50% and CPAP of 5-6 cmH_2_O after INSURE procedure.

Umbilical or peripheral artery catheter was inserted for all patients and ABG according to the patient status was obtained every 30 minutes to 4 hours (OPTI CCA-TS blood gas analyzer, OPTI Medical Company, USA). All patients were monitored continuously, physical examination was done repeatedly, and pulse oximetry was performed by a probe at distal extremity (SpO_2 _Masimo set, NOVIN S1800 patient monitor, Saadat Co., Ltd., Iran). In this study, blood gas analysis was the gold standard. pH higher than 7.20, PaO_2_ equal to 50-80 mmHg, PaCO_2_ less than 60 mmHg, and oxygen saturation in the range of 85-95% were considered as normal values.^7^

Variables including pH, PaO_2_, PaCO_2_, oxygen saturation, as well as gestational age, birth weight, and gender of the infant were recorded. SPSS 19 was used to analyze the data. To assess the degree of dependency among observations of the same patients, we estimated the ICC value by the applied multilevel analysis. Sensitivity, specificity, positive predictive and negative predictive values were extracted from 2×2 contingency tables of results.^8^

In this study, any abnormality in blood gas analysis including low pH, high PaCO_2_, low PaO_2_, and high PaO_2 _was considered as “disease/condition”, and SpO_2_ value by pulse oximetry was considered as “test”***.***

The study protocol received approval from the Ethics Committee of Kerman University of Medical Sciences with the code number 323/90/K, and all patients provided written informed consent prior to participation. 

## Results

Among 30 preterm infants who were studied, 14 (46.7%) were female and 16 (53.3%) were male. Mean gestational age at birth was 31^1/7^-week, with minimum of 27-week and maximum of 35^1/7^-week. Mean birth weight was 1340 grams, with the range of 900 to 1500 grams.

193 ABG specimens were obtained from the infants through umbilical or peripheral arterial catheters. Of the 193 blood samples, only 14 specimens (7.2%) were found to have one or more of acidosis, hypercapnia, and hypoxemia.

Acidosis (pH<7.20) was found in 10 specimens from a total of 193. Sensitivity of pulse oximeter in the estimation of acidosis was 40% and its specificity was 100%. Positive predictive value and negative predictive value of pulse oximetry in the prediction of acidosis were 100% and 96.8%, respectively.

From 193 ABG specimens, hypercapnia (PaCO_2_>60) was found in six specimens. Sensitivity of pulse oximetry with a cut-off point of 85% in the detection of hypercapnia was 50%. Specificity of pulse oximetry in the prediction of normocapnia was 99.6%. Positive predictive value and negative predictive value of pulse oximetry for PaCO_2_ were 75% and 98.4%, respectively.

From 193 specimens, four showed hypoxemia (PaO_2_<50) by blood gas analysis. Sensitivity of pulse oximetry in the detection of hypoxemia was 75% and its specificity was 99.5%. Positive predictive value and negative predictive value of pulse oximetry for hypoxemia were 75% and 98.5%, respectively.

In the evaluation of hyperoxemia (PaO_2_>80), from 193 ABG specimens, 61 had PaO_2_ higher than 80 (hyperoxemia). Sensitivity of pulse oximetry in the detection of hyperoxemia was 83% and its specificity was 92.4%. Positive predictive value and negative predictive value for hyperoxemia were 83% and 92.4%, respectively (table 1).

**Table 1:** Prediction of ABG abnormalities by pulse oximetry in neonates with RDS

	**Acidosis** **(pH<7.20)**	**Hypercapnia** ** (PaCO_2_>60) **	**Hypoxemia** ** (PaO_2_<50) **	**Hyperoxemia** ** (PaO_2_>80) **
Cut-off point SpO_2_ (%)	85	85	85	95
Sensitivity (%)	40	50	75	83
Specificity (%)	100	99.6	99.5	92.4
PPV (%)	100	75	75	83
NPV (%)	96.8	98.4	98.5	92.4

NPV: Negative predictive value; PPV: Positive predictive value

## Discussion

In a study performed by Witting et al., sensitivity and specificity of room-air pulse oximetry (with SpO_2_≥96%) in detecting moderate hypercapnia (PaCO_2_>50) in patients admitted to emergency ward were 96% and 39%, respectively. They concluded that room-air oxygen saturation below 95% could alert the physician to the onset of hypoventilation.^9^

In another study, Ritonga et al*.* assessed the validity of pulse oximetry in the estimation of hypoxemia and hyperoxemia in neonates and children. Pulse oximetry test (with cut-off point 91%) for detecting hypoxemia in neonates (PaO_2_<35 mmHg) had a sensitivity of 81%, and specificity of 79%. Pulse oximetry test with cut-off point 95% for detecting hyperoxemia (PaO_2_>50) had a sensitivity of 78% and specificity of 66%. They concluded that the validity of pulse oximetry in the detection of hypoxemia in neonates was fairly good, but, it was not good enough to be used in the estimation of hyperoxemia in neonates.^10^

In a study performed by Bakr et al*.,* the diagnostic value of fetal pulse oximetry in comparison with fetal scalp blood gas in predicting neonatal outcome was assessed and it was found that these two tests were favorably comparable.^11^

In another study, Carruthers et al*.* compared arterial blood gas analysis with oxygen saturation by pulse oximetry in the assessment of acute asthma. They concluded that, in SpO_2_>92%, respiratory failure is not probable and ABG analysis is not necessary.^12^

In our study, the diagnostic value of pulse oximetry in the detection of hyperoxemia was high. None of the infants with SpO_2_ value greater than 95% were acidotic or hypercapnic. So, one can rely on SpO_2_ values for further management of patients with high SpO_2_ (>95%), which is sensitive for hyperoxemia, and decrease FiO_2_ or CPAP.

The validity of pulse oximetry in predicting hypoxemia in our study was not good. In cases that were hypoxemic according to blood gas, the sensitivity of pulse oximetry was 75%. This means that hypoxemia detection by contenting oneself with pulse oximetry alone; one would miss hypoxemia in 25% of cases.

The sensitivity of pulse oximetry in the detection of acidosis in our study was only 40%. In other words, the prediction of acidosis by pulse oximetry alone will result in missing acidosis in 60% of cases.

In the present study, the sensitivity of pulse oximetry in detecting hypercapnia was 50%. In other words, based on SpO_2_ by simultaneous pulse oximetry alone, half of hypercapnic patients would be missed.

Therefore, in patients with SpO_2_ values lower than 95%, arterial blood gas measurements should be performed, and according to ABG results, increasing FiO_2_ or CPAP, surfactant administration, or initiation of mechanical ventilation should be done.

## Conclusion

Pulse oximetry in the detection of hyperoxemia in neonates <1500 grams with RDS, who are treated according to INSURE protocol, has a good validity to appropriately manage the patient without blood gas analysis. However, the validity of pulse oximetry is not good enough to be used to detect acidosis, hypercapnia, and hypoxemia. 
